# SARS-CoV-2 Infection Risk by Vaccine Doses and Prior Infections Over 24 Months: ProHEpiC-19 Longitudinal Study

**DOI:** 10.2196/56926

**Published:** 2024-11-22

**Authors:** Pere Torán-Monserrat, Noemí Lamonja-Vicente, Anna Costa-Garrido, Lucía A Carrasco-Ribelles, Bibiana Quirant, Marc Boigues, Xaviera Molina, Carla Chacón, Rosalia Dacosta-Aguayo, Fernando Arméstar, Eva María Martínez Cáceres, Julia G Prado, Concepción Violán, ProHEpiC-19 study group, Magda Alemany Costa

**Affiliations:** 1 Unitat de Suport a la Recerca Metropolitana Nord, Institut Universitari d’Investigació en Atenció Primària Jordi Gol, Mare de Déu de Guadalupe, 2, Mataró, 08303, Spain, 34 7415338; 2 Germans Trias i Pujol Research Institute, Badalona, Spain; 3 Department of Medicine, Faculty of Medicine, Universitat de Girona, Girona, Spain; 4 Multidisciplinary Research Group in Health and Society (2021-SGR-0148), Institut Universitari d’Investigació en Atenció Primària Jordi Gol, Mataró, Spain; 5 Grup de REcerca en Impacte de les Malalties Cròniques i les seves Trajectòries (2021 SGR 01537), Institut Universitari d’Investigació en Atenció Primària Jordi Gol, Barcelona, Spain; 6 Immunology Department, Federation of Clinical Immunology Societies Center of Excellence, Universitat Autònoma de Barcelona, Cerdanyola del Vallès, Spain; 7 Immunology Division, Laboratori Clinic Metropolitana Nord, Hospital Universitari Germans Trias i Pujol, Badalona, Spain; 8 Department of Medicine, Universitat Autònoma de Barcelona, Cerdanyola del Vallès, Spain; 9 Programa de Màster en Salud Pública, Universitat Pompeu Fabra, Barcelona, Spain; 10 Centro de Epidemiología y Políticas de Salud, Universidad del Desarrollo, Santiago de Chile, Chile; 11 Intensive Care Unit, Hospital Universitari Germans Trias i Pujol, Badalona, Spain; 12 IrsiCaixa-AIDS Research Institute, Badalona, Spain; 13 Centro de Investigación Biomédica en Red de Enfermedades Infecciosas, Instituto de Salud Carlos III, Madrid, Spain; 14 Grup de Recerca en Impacte de les Malalties Cròniques i les seves Trajectòries (2021-SGR-01537), Germans Trias i Pujol Research Institute, Badalona, Spain; 15 Red de Investigación en Cronicidad, Atención Primaria y Prevención y Promoción de la Salut, Instituto de Salud Carlos III, Madrid, Spain; 16 see Acknowledgments

**Keywords:** SARS-CoV-2, COVID-19, health care workers, cohort, extended Cox models, coronavirus, epidemiology, epidemiological, risks, infectious, respiratory, longitudinal, vaccines, vaccination, vaccinated

## Abstract

**Background:**

As the vaccination campaign against COVID-19 progresses, it becomes crucial to comprehend the lasting effects of vaccination on safeguarding against new infections or reinfections.

**Objective:**

This study aimed to assess the risk of new SARS-CoV-2 infections based on the number of vaccine doses, prior infections, and other clinical characteristics.

**Methods:**

We defined a cohort of 800 health care workers in a 24-month study (March 2020 to December 2022) in northern Barcelona to determine new infections by SARS-CoV-2. We used extended Cox models, specifically Andersen-Gill (AG) and Prentice-Williams-Peterson, and we examined the risk of new infections. The AG model incorporated variables such as sex, age, job title, number of chronic conditions, vaccine doses, and prior infections. Additionally, 2 Prentice-Williams-Peterson models were adjusted, one for those individuals with no or 1 infection and another for those with 2 or 3 infections, both with the same covariates as the AG model.

**Results:**

The 800 participants (n=605, 75.6% women) received 1, 2, 3, and 4 doses of the vaccine. Compared to those who were unvaccinated, the number of vaccine doses significantly reduced (*P*<.001) the risk of infection by 66%, 81%, 89%, and 99%, respectively. Unit increase in the number of prior infections reduced the risk of infection by 75% (*P*<.001). When separating individuals by number of previous infections, risk was significantly reduced for those with no or 1 infection by 61% (*P*=.02), and by 88%, 93%, and 99% (*P*<.001) with 1, 2, 3, or 4 doses, respectively. In contrast, for those with 2 or 3 previous infections, the reduction was only significant with the fourth dose, at 98% (*P*<.001). The number of chronic diseases only increased the risk by 28%‐31% (*P*<.001) for individuals with 0‐1 previous infections.

**Conclusions:**

The study suggests that both prior infections and vaccination status significantly contribute to SARS-CoV-2 immunity, supporting vaccine effectiveness in reducing risk of reinfection for up to 24 months after follow-up from the onset of the pandemic. These insights contribute to our understanding of long-term immunity dynamics and inform strategies for mitigating the impact of COVID-19.

## Introduction

As the COVID-19 vaccination campaign progresses, it is important to understand the lasting effects of vaccination in preventing new infections or reinfection. Studies have shown protective effects of COVID-19 vaccination against severe disease and mortality, but they have not provided clear evidence of the lasting effects of vaccination in preventing new infections or reinfection as the campaign progresses [1-4].

Reinfection occurs when an individual is infected with SARS-CoV-2, recovers, and then becomes infected again after some time. According to the Centers for Disease Control and Prevention [5], reinfections can occur multiple times and, although they are most often mild, severe illnesses can also occur. The risk of reinfection can vary based on demographic characteristics, vaccination history, and risk exposure. The risk of reinfection is also influenced by viral evolution and the emergence of new viral variants that can evade preexisting immunity. It is noteworthy that even if an individual gets reinfected, their immune response should protect them from severe illness and hospitalization [6] and that, in general, reinfections have been reported to be less clinically severe than initial SARS-CoV-2 infections. The chances of reinfection are lower if an individual is fully vaccinated and receives subsequent booster doses [7,8]. Breakthrough infections (BTIs) are infections produced after a SARS-CoV-2 complete vaccination (2 doses) [9].

Despite multiple studies conducted on reinfections and BTIs, these have primarily focused on studying symptoms, periods of reinfection, and other health outcomes [10-15]. However, little is known about the impact of the number of vaccinations and prior infections on the risk of new SARS-CoV-2 infections. The initial studies on SARS-CoV-2 reinfections focused more on attempting to confirm reinfection by different genetic strains of SARS-CoV-2 [16]. Another study analyzed the severity of reinfections in a cohort of health care workers [17]. A recent review found that studies tended to focus on the characteristics and risk factors of SARS-CoV-2 reinfection, particularly during the Omicron wave, but did not compare the severity of reinfection and primary infection before vaccine availability. This review suggested that reinfections are generally milder than primary infections, with lower viral loads and fewer self-reported symptoms. However, they did not specifically address the relative severity of reinfections versus primary infections in the early period of the pandemic, before vaccines became available [18].

Addressing this question requires the application of extended Cox models that allow the analysis of correlated recurrent events. The Andersen-Gill (AG) model assumes a common baseline hazard function, meaning that the baseline risk remains the same for each event (eg, the first, second, or third infection). Given this assumption, it is appropriate to use either a stratified model such as the Prentice-Williams-Peterson (PWP) model, which stratifies by the number of infections, or an AG model that is adjusted for a time-dependent variable. Both approaches are suitable for analyzing recurrent events [19]. To apply these survival models, it is necessary to have a well-characterized cohort of individuals, such as the Professionals’ Health in Epidemiological Crises COVID-19 (ProHEpiC-19) cohort. This cohort provides a well-characterized population of health care workers (HCWs) to investigate the multiple determinants of risk of new infection [20]. Our findings are anticipated to contribute to the ongoing efforts to optimize vaccination strategies, enhance long-term protection, and mitigate the impact of COVID-19. Ultimately, knowledge derived from this research can help shape evidence-based public health interventions and guide the response to vaccination campaigns. Our findings are expected to contribute to ongoing efforts to optimize vaccination strategies, improve long-term protection, and mitigate the impact of COVID-19. In this context, the main objective of our study is to assess the risk of SARS-CoV-2 infections (including first infections, reinfections, and BTIs) by examining the number of vaccine doses received, prior infections, and other clinical characteristics over 24 months of follow-up.

## Methods

### Study Design, Participant Recruitment, and Follow-Up

The ProHEpiC-19 cohort is a prospective, longitudinal study of HCWs in the Northern Metropolitan Area of Barcelona, Spain, including physicians, nurses, nursing assistants, researchers, and other essential workers in direct contact with patients during the first, second, and successive waves of the COVID-19 pandemic. The cohort participants were recruited between March 3, 2020, and March 22, 2022, with the following inclusion criteria: (1) aged *>*18 years and (2) agreed to take part in the study, agreed to participate, and signed the informed consent. The exclusion criteria included those who did not accept participation in the study and/or did not sign the informed consent.

**Figure 1.**
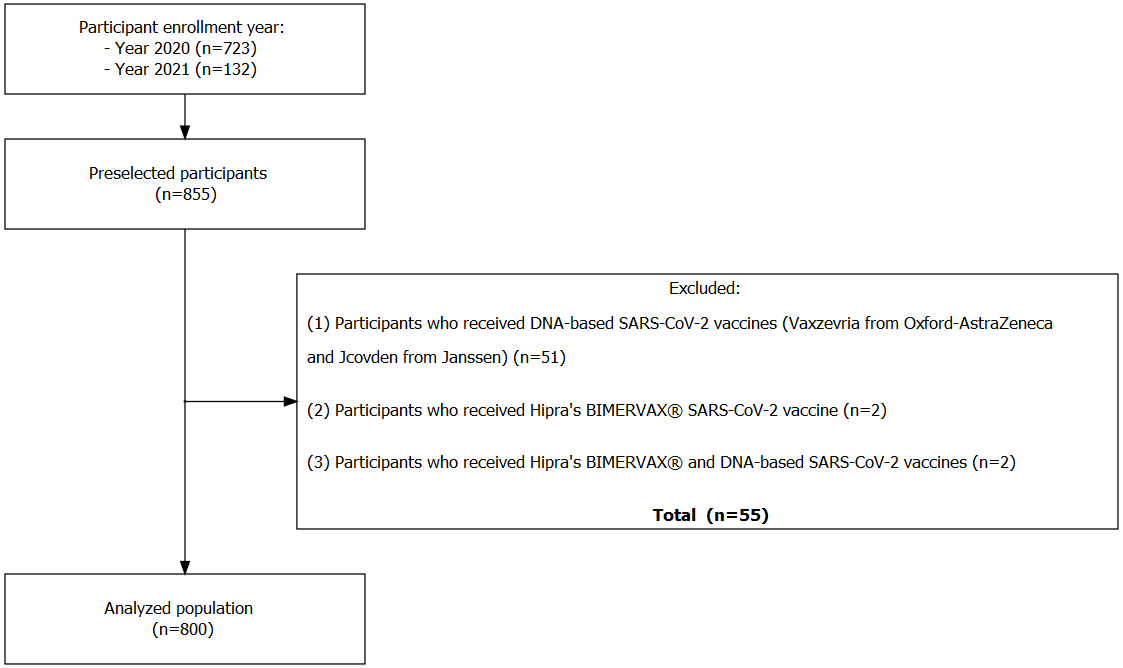
Flowchart of the study population. The figure reports the participant’s entry into the study, the number of individuals who met each exclusion criterion, and the number of individuals that met all the inclusion criteria.

All participants completed the clinical questionnaires and were examined for COVID-19–specific symptoms at the first visit at study entry (baseline) and follow-up visits (7, 15, 30, 60, 90, 180, and 270 days, and 12, 18, and 24 months after baseline). In addition, we performed reverse transcription polymerase chain reaction (RT-PCR) tests for nasal and oropharyngeal swabs and antibody tests or antigen-detection rapid diagnostic tests (Ag-RDTs) for nasal swabs at the first and second visit (baseline and 7 days). The Ag-RDTs were repeated at 7, 15, 30, 60, 90, 180, and 270 days, and 12, 18, and 24 months after the baseline (Figure 1).

### Data Sources

We used 2 data sources. The first was a database obtained from electronic health records (EHR) in the Catalan public health system that provides an electronic clinical record, facilitating comprehensive patient management. This system supports clinical decision-making with high safety and quality of care. The information gathered includes SARS-CoV-2 vaccination details, results from RT-PCR tests and Ag-RDTs, diagnoses, and drug prescriptions. Second, REDCap (version 12.4.22; Vanderbilt University) was utilized to ensure the privacy of our participants by implementing data collection specific to the study. This includes unique numeric patient identifiers, and demographic, social, and clinical data of professionals obtained from questionnaires during follow-up. The unique numeric patient identifier allows for linking the 2 data sources.

### SARS-CoV-2 Detection and Quantification of Immunoglobulin G and Immunoglobulin M

The RNA for RT-PCR testing was extracted from fresh samples using the STARMag 2019-nCoV kit (Seegene Inc) by means of a liquid-dispensing robot. The SARS-CoV-2 RNA was detected using the Allplex SARS-CoV-2 assay, a multiplex RT-PCR assay that detects 4 SARS-CoV-2 target genes in a single tube. Qualitative assays were performed. For the antibody tests, we conducted a prevalidation study with 6 different commercially available and in vitro diagnostic CE-approved enzyme-linked immunosorbent assay (ELISA) tests and selected anti–SARS-CoV-2 immunoglobulin (Ig) G (anti-N) and IgM (anti-N) by ELISA kits based on their performance. Infected participants were also tested for their total levels of antibodies against the spike (S) subunit of SARS-CoV-2 using the DECOV1901 ELISA kit (Demeditic Diagnostic GmbH), which allows quantitative measurement of total IgG. The Ag-RDTs used were those that had been previously validated [21].

### Variables

#### Outcomes

Study outcomes were as follows:

1. The first SARS-CoV-2 infection was detected by positive RT-PCR, Ag-RDTs, IgG (anti-N), IgM (anti-N), or IgG (anti-S) test before the first dose of SARS-CoV-2 vaccine. The presence of symptoms complemented these criteria before the baseline visit (collected through the questionnaires) and a positive basal IgG (anti-N), IgM (anti-N), or IgG (anti-S) result before the first dose of the SARS-CoV-2 vaccine. In such cases, the date of infection was considered the date of the serological test. Additionally, diagnoses of SARS-CoV-2 after April 2022 were added to the EHR database.
2. New infection refers to cases of SARS-CoV-2 infection after being notified of a first infection. Two positive RT-PCR tests or Ag-RDTs with a 3-month gap in between were required to detect new infections. These new infections were recorded as the date of the second positive RT-PCR test or Ag-RDT. We also took into account infections that occurred more than 14 days after the first dose and more than 7 days after the second or additional doses of the vaccine.

#### Covariates

At baseline, various variables were collected: (1) sociodemographic information, including sex, age (categorized as ≤29, 30-39, 40-49, ≥50 years or as ≥10-year increase, depending on the analysis), and job title (categorized as physician, nurse, nurse assistant, or others, including physiotherapists, management and administrative staff, and social workers); (2) smoking status, categorized as never smoker, former smoker, or smoker; and (3) chronic conditions (all were obtained in primary care, using International Classification of Diseases, 10th revision). Chronic diseases were measured using the operational definition of the Swedish National Study of Aging and Care in Kungsholmen (SNAC-K), which defined 60 categories of chronic conditions using more than 900 International Classification of Diseases, 10th revision codes, along with clinical, laboratory, and drug-related parameters for assessing certain conditions [22,23]. The number of chronic conditions per participant was obtained and categorized as 0‐1, 2‐3, and >3. Moreover, the 5 most prevalent diagnoses were obtained, as well as medications dispensed in pharmacies (using Anatomical Therapeutic Classification, fifth level). The number of drugs prescribed per participant was obtained and categorized as 0‐1, 2‐3, and >3. Additionally, the 5 most prevalent types of drugs prescribed were obtained.

During follow-up, the following variables were collected: (1) SARS-CoV-2 vaccines—participants received the mRNA-based vaccines BNT162b2 (Pfizer-BioNTech) or mRNA-1273 COVID-19 (Moderna)—and the number of doses per participant were recorded and categorized as 1, 2, 3, or 4; and (2) number of SARS-CoV-2 infections, treated as a numerical variable.

### Statistical Analysis

We conducted a descriptive analysis of baseline characteristics for all participants (n=800). All variables were considered categorical and presented using frequencies and percentages.

Another descriptive analysis was performed to compare the baseline characteristics between participants with 0‐1 and 2‐3 infections. All variables were treated as categorical and the *χ*^2^ test was used for statistical evaluation.

To model recurrent SARS-CoV-2 infections, extended Cox models were used, as these models can handle multiple events within the same subject. Specifically, the study utilized 2 types of extended Cox models: the AG model [24] and the PWP model [25]. The AG model assumes that the risk of an event is unaffected by prior events, using a common baseline risk function for all occurrences. In contrast, the PWP model is stratified by the number of previous events, meaning each event has a distinct risk and is conditional on the occurrence of the preceding event.

The choice between these models depends on the nature of the recurrent infection process. If subsequent infection risk changes with the number of previous infections, a model incorporating time-dependent covariates (adjusted AG model) or separate strata for each event (PWP model) is recommended. If the risk remains constant, an unadjusted AG model is suitable.

To mitigate potential inflation of type I errors due to multiple observations per individual, the analysis used the sandwich robust standard error method. We found that the risk of infection decreased with more prior infections, suggesting either a stratified or an adjusted AG model was appropriate [26,27].

Both the AG and PWP models were adjusted for sociodemographic factors, chronic conditions, vaccination status, and infection count. Statistical significance was set at *P*<.05 and analyses were performed using R (R Foundation for Statistical Computing) and RStudio (Posit PBC). For more information, see Statistical Methods in Multimedia Appendix 1.

### Ethical Considerations

The ethics committees of the Foundation University Institute for Primary Health Care Research Jordi Gol i Gurina (reference number 20/067, reference number 21/032-PCV, and reference number 22/093-PCV) and The Germans Trias i Pujol Research Institute (reference number COV20/00660 [PI-20-205]) approved the study protocol. All methods were carried out in accordance with the institutional guidelines and regulations and were conducted in accordance with the ethical standards of the Declaration of Helsinki. All participants recruited in the study were fully informed about the ProHEpiC-19 protocol and signed informed consent forms to participate.

## Results

### Participant Characteristics

This study involved 800 participants, 75.6% (n=605) of whom were female. Among them, 35.5% (n=284) were ≥50 years old, 30% (n=240) were nurses, and 64.3% (n=499) were nonsmokers. HCWs with 2‐3 and >3 chronic conditions accounted for 29.5% (n=236) and 25% (n=200) of the cohort, respectively. The 2 most common chronic conditions were neurotic, stress, and somatoform diseases (n=162, 20.2%) and obesity (n=101, 12.6%). Prescription of drugs with 2‐3 and >3 types affected 11.6% (n=93) and 5.3% (n=42) of HCWs, respectively. The 2 most prescribed drugs were for other musculoskeletal joint diseases (n=110, 13.8%) and colitis-related diseases (n=102, 12.8%; Table 1).

The relationship between participant characteristics and the number of infections was investigated. The predominant groups included individuals aged 40‐49 years, other health care professionals, participants with no or 1 chronic disease, and individuals who received 2‐3 doses of the vaccine (Table 2).

**Figure 2.**
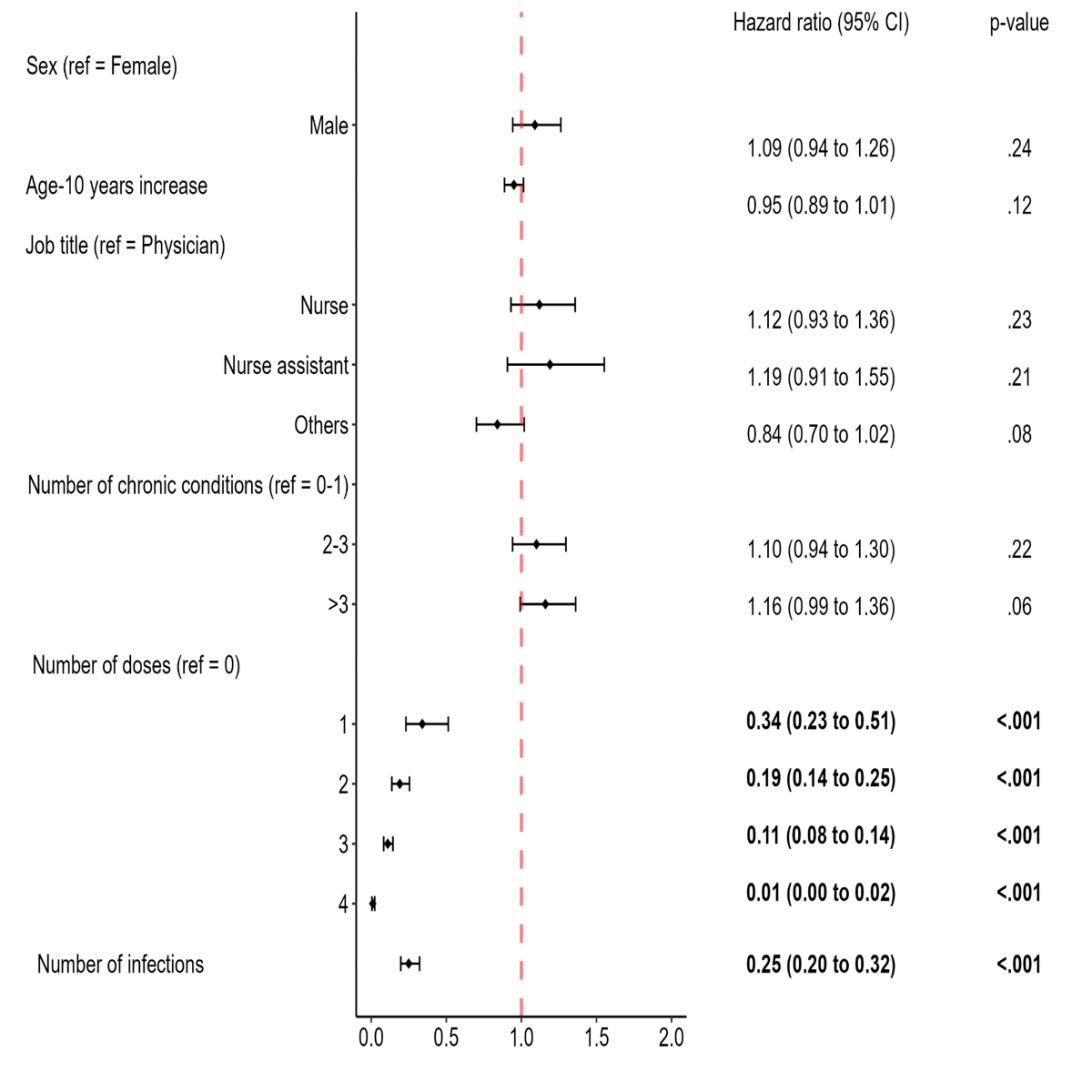
SARS-CoV-2 infection hazard ratios as calculated by the Andersen-Gill model. Note: The model uses a common baseline risk function and accounts for a time-dependent variable (number of infections). The number of chronic diseases was calculated using the operational definition of the Swedish National Study of Aging and Care in Kungsholmen. Statistical significance (*P*<.05) is denoted in bold. Ref: reference group.

Figure 2 presents the results of the AG model, which was adjusted for the time-dependent variable encoding the number of infections. Participants who received 1, 2, 3, and 4 doses of the vaccine had a significantly lower infection risk than those without any dose. The fitted model estimates that individuals with 1, 2, 3, and 4 doses of vaccine reduce the risk of infection by 66%, 81%, 89%, and 99%, respectively.

**Table 1.** Sociodemographic and clinical characteristics of all participants. Note: All characteristics were collected at the study’s baseline except for type of primary infection, which was obtained at the end of the follow-up. The number of chronic diseases (using the operational definition of the Swedish National Study of Aging and Care in Kungsholmen), the prevalence of the 5 most prevalent conditions, and the number of medications dispensed (using the Anatomical Therapeutic Chemical, fifth level) and the 5 most prevalent types are described.

	Participants (N=800), n (%)
**Sex (female)**	605 (75.6)
**Age (years)**	
≤29	112 (14)
30‐39	149 (18.6)
40‐49	255 (31.9)
≥50	284 (35.5)
**Job title**	
Physician	216 (27)
Nurse	240 (30)
Nurse assistant	65 (8.1)
Others	279 (34.9)
**Smoking status**	
Never smoker	499 (64.3)
Former smoker	143 (18.4)
Smoker	134 (17.3)
**Number of chronic conditions**	
0‐1	364 (45.5)
2‐3	236 (29.5)
>3	200 (25)
**Neurotic, stress, and somatoform diseases**	162 (20.2)
**Obesity**	101 (12.6)
**Dyslipidemia**	100 (12.5)
**Migraine facial pain syndrome**	98 (12.2)
**Dorsopathies**	97 (12.1)
**Number of drugs**	
0‐1	665 (83.1)
2‐3	93 (11.6)
>3	42 (5.2)
**Drug for other musculoskeletal joint diseases**	110 (13.8)
**Drug for colitis-related disease**	102 (12.8)
**Drug for neurotic, stress, and somatoform diseases**	93 (11.6)
**Drug for peripheral neuropathy**	91 (11.4)
**Drug for dorsopathies**	85 (10.6)
**Type of primary infection**	
Not infected	69 (8.6)
With reverse transcription polymerase chain reaction or antigen detection rapid diagnostic test	572 (71.5)
With IgG^a^ (anti-N), IgM (anti-N), or IgG (anti-S)	62 (7.8)
Had symptoms before first visit and a positive basal IgG (anti-N), IgM (anti-N), or IgG (anti-S)	49 (6.1)
Diagnoses from the electronic health record database	48 (6)

a Ig: immunoglobulin.

**Table 2.** Sociodemographic and clinical characteristics of all participants stratified by the number of SARS-CoV-2 infections each participant had at the end of the follow-up. Note: All characteristics were collected at the study‘s baseline except for the number of vaccine doses, which was obtained at the end of the follow-up. The number of chronic diseases (using the operational definition of the Swedish National Study of Aging and Care in Kungsholmen study), the prevalence of the 5 most prevalent conditions, and the number of medications dispensed (using the Anatomical Therapeutic Classification, fifth level) and the 5 most prevalent types are described.

	With no or 1 infection (n=595), n (%)	With 2 or 3 infections (n=205), n (%)	Chi-square (*df*)	*P* value
**Sex (female)**	447 (75.1)	158 (77.1)	0.217 (1)	.64
**Age (years)**			13.386 (3)	.004
≤29	73 (12.3)	39 (19)		
30‐39	108 (18.2)	41 (20)		
40‐49	183 (30.8)	72 (35.1)		
≥50	231 (38.8)	53 (25.9)		
**Job title**			16.092 (3)	.001
Physician	165 (27.7)	51 (24.9)		
Nurse	162 (27.2)	78 (38)		
Nurse assistant	42 (7.1)	23 (11.2)		
Others	226 (38)	53 (25.9)		
**Smoking status**			0.567 (2)	.75
Never smoker	374 (64.7)	125 (63.1)		
Former smoker	103 (17.8)	40 (20.2)		
Smoker	101 (17.5)	33 (16.7)		
**Number of chronic conditions**			7.199 (2)	.03
0‐1	256 (43.0)	108 (52.7)		
2‐3	178 (29.9)	58 (28.3)		
>3	161 (27.1)	39 (19)		
**Number of drugs**			2.564 (2)	.28
0‐1	488 (82)	177 (86.3)		
2‐3	72 (12.1)	21 (10.2)		
>3	35 (6)	7 (3.4)		
**Number of doses**			10.623 (4)	.03
0	19 (3.2)	6 (2.9)		
1	33 (5.6)	18 (8.8)		
2	121 (20.3)	57 (27.8)		
3	285 (47.9)	92 (44.9)		
4	137 (23)	32 (15.6)		

Moreover, the time-dependent variable was also significant; the hazard ratio for “number of infections” was 0.25. This means that each additional previous infection, in combination with the other variables, reduced the infection risk 75%.

Finally, we found that sex, age, job title, and number of chronic conditions were not statistically significant factors associated with the risk of infection. Table S1 in Multimedia Appendix 1 reports the hazard ratios for the same AG model but with a more detailed job title description. None of them had a significantly different hazard ratio.

**Figure 3.**
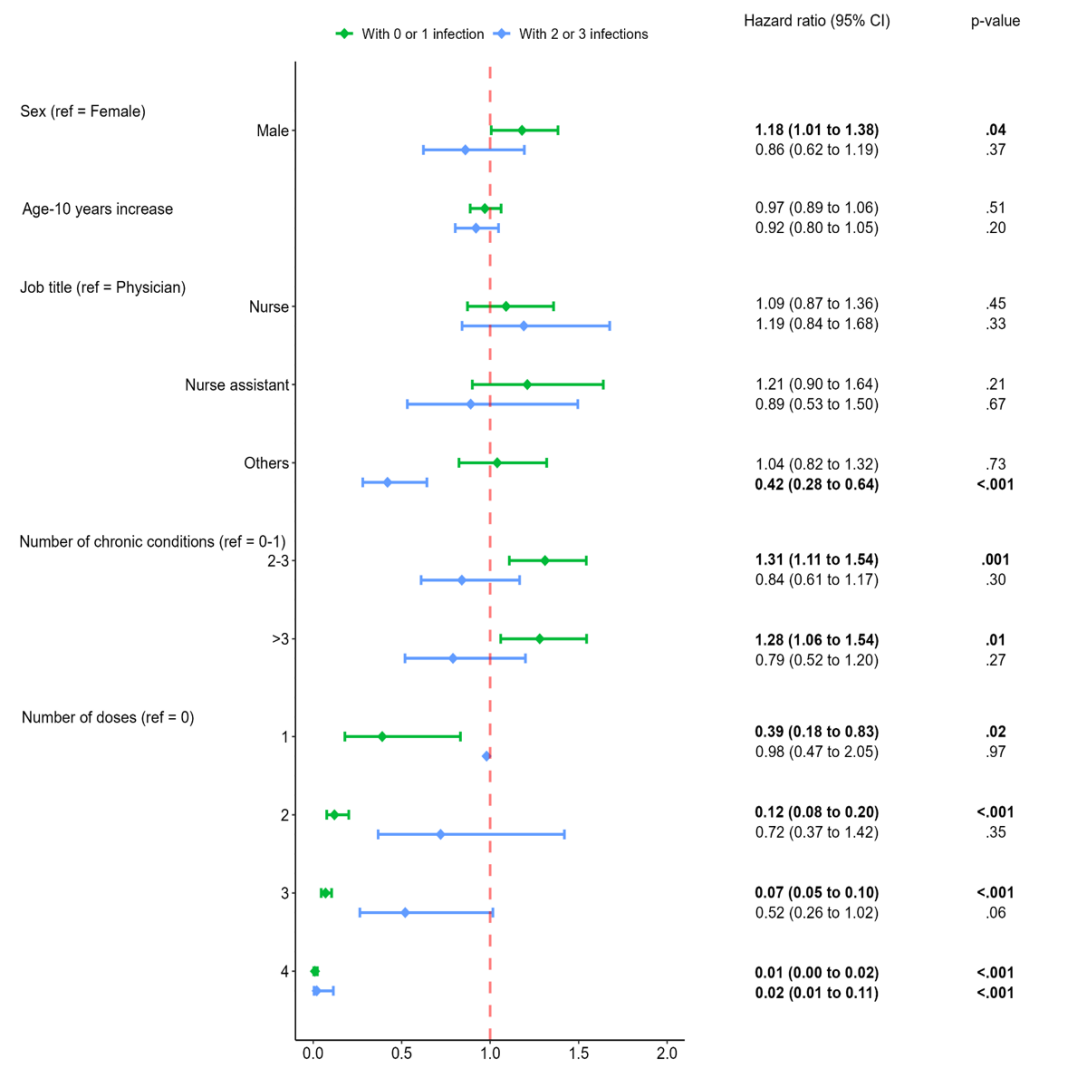
SARS-CoV-2 infection hazard ratios as calculated by the Prentice-Williams-Peterson models. Note: One of the models includes the participants with no or 1 infection, and the other those with 2 or 3 infections. The number of chronic diseases was calculated using the operational definition of the Swedish National Study of Aging and Care in Kungsholmen. Statistical significance (*P*<.05) is denoted in bold. Ref: reference group.

Two PWP models were adjusted: one included subjects without or with a single infection, and the other model involved subjects with 2 or 3 infections. In both models, participants who received 4 doses had a significantly lower risk of infection than those without any doses. However, in the model with no or 1 infection, there was a reduced risk of infection with 1, 2, and 3 doses, leading to risk reductions of 61%, 88%, and 93%, respectively (Figure 3).

In the model accounting for 2 or 3 infections, job title was associated with the risk of infection. Individuals categorized under the “others” job title exhibited a decreased risk of infection by 58%, in contrast to those belonging to the physicians category. Table S2 in Multimedia Appendix 1 reports the hazard ratios for the same PWP models but with a more detailed job title description. Physiotherapists and other professionals showed a significantly lower risk, but only for participants with 2 or 3 infections.

Last, in the model with no or 1 infection, having 2‐3 and >3 chronic conditions increased the risk of infection by 31% and 28%, respectively. In addition, being male was associated with an increased risk of infection compared to being female (Figure 3).

## Discussion

### Principal Findings

Our study spanning 24 months of follow-up, conducted within a cohort of 800 HCWs, analyzed new SARS-CoV-2 infections since the onset of the SARS-CoV-2 pandemic, taking into account prior infections, vaccination, and other clinical or phenotypic characteristics.

Our results point out 3 key aspects regarding the risk of presenting with new SARS-CoV-2 infections. First, participants who received 1, 2, 3, and 4 doses of the vaccine had a significantly lower risk of infection compared to those without any doses. The AG model estimated a reduction in infection risk of 66%, 81%, 89%, and 99%, respectively. Second, the risk of infection decreased by 75% as the number of prior infections increased. Third, for participants with no or 1 infection, apart from showing a reduction in infection after receiving any vaccine dose, having 2‐3 and >3 chronic conditions and being male increased the risk of infection. In contrast, for participants with a history of multiple infections (2 or 3), the risk was influenced only by the fourth vaccine doses and specific job titles; particularly those categorized as “others” exhibited a decreased risk compared to those belonging to the physicians category. The job title emerged as a significant factor influencing infection risk. Those categorized under the “others” job title had a 58% lower risk of infection compared to physicians. This discrepancy could be attributed to differing levels of exposure, variations in adherence to preventive measures, or inherent occupational risks associated with different job titles. The identification of job title as a risk factor suggests that targeted interventions and protective strategies should be tailored to specific occupational groups to mitigate infection risk effectively [28].

Regarding PWP models, one model focused on subjects with no or 1 infection and the other on those with 2 or 3 infections. The results consistently demonstrated the protective effect of vaccination, as subjects who received 4 doses exhibited a significantly lower risk of infection across both models. These findings highlight the dose-response relationship between vaccination and infection risk reduction, underscoring the importance of complete vaccination regimens. The analysis also revealed that chronic conditions and gender were significant predictors of infection risk in the model with no or 1 infection. Individuals with 2-3 chronic conditions and those with more than 3 chronic conditions experienced a 31% and 27% increased risk of infection, respectively. This association underscores the heightened vulnerability to infection of individuals with multiple chronic conditions, likely due to compromised immune function or the presence of comorbidities that exacerbate infection susceptibility.

These findings advocate for prioritizing vaccination and other preventive measures for individuals with chronic conditions to reduce their infection risk.

Moreover, gender differences in infection risk were observed, with males exhibiting a higher risk of infection compared to females. This gender disparity could be influenced by behavioral, biological, or social factors, including differences in immune response, health-seeking behaviors, or occupational exposures. Understanding these gender-specific risk factors is crucial for developing targeted public health interventions to address the unique needs of different demographic groups.

The results underline the importance of vaccination in mitigating the risk of infection and highlight the dose-dependent relationship between the number of vaccine administrations and protection against SARS-CoV-2 infection and reinfection. These data corroborate that SARS-CoV-2 vaccination promotes early activation of memory T cells and enhances immune responses during BTIs [29,30]. These memory T cells are capable of robust recall responses, both humoral and cellular, following booster vaccination, supporting the functional nature of mRNA vaccine–induced immune memory [31]. Additionally, SARS-CoV-2 BTI induces rapid and extensive recall of memory T cell populations, with spike-specific CD4+ and CD8+ T cell activation occurring early and favoring viral clearance [32].

In addition, our findings pointed out the importance of vaccination in previously infected participants and this aligns with previous studies that have emphasized the significance of hybrid immunity (ie, the combination of SARS-CoV-2 infection and vaccination) to offer long-term immune responses and the highest rate of protection against SARS-CoV-2 [33].

Several studies have investigated the effects of SARS-CoV-2 vaccination in previously infected people. The analysis of hybrid immunity since the beginning of the pandemic with the survival models applied has allowed us to observe that the reduction in the risk of infection in previously infected people depends on the number of vaccines administered and the number of previous infections. Furthermore, our results corroborate findings from immunological studies that hybrid immunity, a combination of prior SARS-CoV-2 infection and vaccination, provides better protection against Omicron infection than vaccine-induced immunity alone [34-36]. These results contrast with other studies. A study found that hybrid immunity confers better protection against SARS-CoV-2 Omicron infection than vaccine-induced immunity [35]. Nevertheless, this effect does not seem to depend on the sequence or number of immunizing events [37]. Another study also highlighted the benefit of vaccination after previous SARS-CoV-2 infection, particularly in the Omicron era. However, that study only analyzed data from the first 3 doses of the vaccine, with a maximum follow-up of 12 months after vaccination [34]. Studies analyzing the characteristics of SARS-CoV-2 reinfections and BTIs have observed that there is a risk of reinfection among individuals previously diagnosed with COVID-19 [38]. Vaccination may lead to higher titers of neutralizing antibodies compared to SARS-CoV-2 infection [39,40], and individuals infected with SARS-CoV-2 can benefit from vaccination, particularly in preventing more transmissible variants [41].

To the best of our knowledge, there are no other studies with such an extensive follow-up period on first infections, reinfections, and BTIs that apply survival models allowing analysis of recurrent events, wherein the time variable is adjusted to analyze the effect of vaccines and prior infections on the occurrence of new episodes of SARS-CoV-2. Previous studies differ from our study in the duration of the study, the design of the study, and the statistical models applied, making it difficult to make comparisons. Nonetheless, some studies provide results in our direction. In a study conducted in Denmark using EHR data, it was observed that in previously infected individuals, the administration of a complete vaccination regimen (2 doses) was significantly associated with a lower incidence of new SARS-CoV-2 infections (against reinfection) compared to individuals who had not received any vaccination. However, vaccination offers lower protection against reinfection with the Omicron variant [42]. Despite this, later doses administered in this study were modified to the Omicron variant and this could influence the results of the study.

Another important aspect to note is that in most studies, information about the predominant variant at that time is not available. Therefore, reinfections may be influenced not only by the response of neutralizing antibodies, but also by the prevalent variant at that moment, which might impact the extent of reinfections postvaccination. Therefore, a standardized surveillance protocol is necessary for reporting suspected BTIs and for better evaluating the implication of emergent viral variants. Studies on BTIs could be instrumental in understanding the neutralizing response to SARS-CoV-2 infection and the corresponding immunity. However, the absence of systematic genomic sequencing of positive SARS-CoV-2 cases worldwide hampers progress in public health surveillance to manage the pandemic globally [43]. New research, including a genetic comparison of SARS-CoV-2 strains, would be beneficial in understanding the frequency and pathophysiology of SARS-CoV-2 reinfections. Although COVID-19 vaccines have proven highly effective, the possibility of BTIs remains a reality, particularly in the context of concerning, continuously emerging variants [13].

Regarding the increased risk in individuals with more than 3 chronic diseases, this has been demonstrated in other studies [44-46].

### Strengths and Limitations

This study has several notable strengths. First, it used a prospective design, allowing for the collection of data over time, which enhanced the validity of the findings. Second, the application of extended Cox models such as AG or PWP models offered the possibility of handling recurrent events, like SARS-CoV-2 infections, within each subject [19]. The AG model, known for its flexibility and parsimony, outperforms other models like the marginal means/rates model, providing a more suitable approach for addressing the research questions. Furthermore, it is applicable for scenarios involving nonconstant but proportional hazard risk; additionally, it uses a common baseline risk function for all events, estimating a global parameter for the factors of interest [47-49]. The PWP model, on the other hand, is an alternative approach that accommodates ordered recurrent events, allowing for event-specific baseline risk functions. This flexibility proves useful when estimating overall effects or event-specific effects for each covariate [49,50], preserving the order of sequential events, and effectively incorporating event dependence [49]. In summary, our model choice depended on the nature of the disease. We observed a lower risk of infection with increased numbers of prior infections, suggesting that both using a stratified model (PWP model) based on infection count and using an AG model adjusted for the same time-dependent variable were equally appropriate [19]. Third, the motivation of HCWs to collect their health data should also be regarded as a strength of the study.

Interpretation of these results should consider the limitations of the study. First, the majority of the ProHEpiC-19 sample is of White European origin, so findings might not apply to other ethnic groups. Future work could broaden the sample recruitment to people more diverse in these respects to extend the generality of the findings. Second, not having measurements of neutralizing antibodies or T cells or genomic sequencing limits the interpretation of some results. However, these determinations are not routinely applicable to such a large number of samples due to the complexity and cost of the determinations. Third, these findings should be interpreted with caution because the conclusions cannot be extrapolated to specific groups at increased risk of developing severe disease, such as older and immunosuppressed people, who were not analyzed in this study.

Thus, the PWP models used in this study provided valuable insights into the factors influencing infection risk among different subject groups. The protective effect of vaccination, the impact of job title, the role of chronic conditions, and gender differences in infection risk were all highlighted as significant determinants. These findings emphasize the need for comprehensive vaccination strategies, targeted interventions for high-risk occupational groups, and tailored public health measures for individuals with chronic conditions and varying gender-specific vulnerabilities.

Future research should continue to explore these factors to refine and optimize infection prevention strategies. Moreover, simple, harmless, and inexpensive pharmaceutical interventions (such as nasal irrigation with salt water) may also be used as preexposure or postexposure prophylaxis to prevent and control the spread of COVID-19 in high risk settings (eg, care homes) [28].

### Conclusion

The findings of this study suggest that both prior infections with SARS-CoV-2, regardless of the variant, and vaccination play a significant role in providing immunity against SARS-CoV-2. Thus, we provide crucial evidence supporting the effectiveness of vaccination against SARS-CoV-2 in reducing the risk of infection over different periods, up to 24 months from the onset of the pandemic.

In this cohort of HCWs, we observed that the administration of 4 vaccine doses decreased the risk of new infections by SARS-CoV-2, with the risk decreasing as the number of vaccine doses administered increased.

Although the objective of this study was not to analyze transmissibility, it provides new evidence on the effect of vaccines in reducing the number of new infections. However, the impact of vaccination on the transmissibility of SARS-CoV-2 needs to be further elucidated. There is growing evidence that vaccination status should not replace mitigation practices such as mask-wearing, physical distancing, and contact-tracing investigations, even within highly vaccinated populations.

These results indicate that the public health policies implemented have been correct and hold practical implications for future outcomes. It will be necessary to continue emphasizing vaccination campaigns and monitoring infection rates in vaccinated populations.

## Supplementary material

10.2196/56926 Multimedia Appendix 1 Statistical methods.

10.2196/56926 Multimedia Appendix 2 List of ProHEpiC-19 Investigators.

## Abbreviations

AG: Andersen-Gill
Ag-RDT: antigen-detection rapid diagnostic test
BTI: breakthrough infection
EHR: electronic health record
ELISA: enzyme-linked immunosorbent assay
HCW: health care worker
Ig: immunoglobulin
ProHEpiC-19: Professionals’ Health in Epidemiological Crises COVID-19
PWP: Prentice-Williams-Peterson
RT-PCR: reverse transcription polymerase chain reaction
