# Supplementary material for: SARS-CoV-2 Infection Risk by Vaccine Doses and Prior Infections Over 24 Months: ProHEpiC-19 Longitudinal Study
Source: JMIR Public Health Surveill. 2024 Nov 22;10:e56926. doi: 10.2196/56926 (PMC11606241; doi:10.2196/56926)
Supplement: Multimedia Appendix 1 [file publichealth-v10-e56926-s001.docx]

**Supplementary Material**

**Statistical methods**

This study conducted a descriptive analysis of baseline characteristics for all participants (n = 800). All variables were considered categorical and presented using frequencies and percentages.

Another descriptive analysis was performed to compare the baseline characteristics between participants with 0-1 and 2-3 infections. All variables were treated as categorical and the chi-square test was employed for statistical evaluation.

The extended Cox models were used to handle recurrent events, specifically SARS-CoV-2 infections, within each subject. Unlike the traditional Cox model, which is designed for modeling a single event, the extended Cox models allows for the analysis of multiple occurrences of events for each individual. Specifically, we considered two types of extended Cox models: the counting process model, also known as the Anderson-Gill (AG) model [1], and the conditional model, referred to as the Prentice-Williams- Peterson (PWP) [2].

AG model proposes an extension of the Cox model where the risk of an individual having an event, at time t, is unaffected by any earlier event. It uses a common baseline risk function for all events and estimates a global parameter for the factors of interest. In this way, it implies that the time increments between events are not correlated. Specifically, they propose to model the risk for the kth infection under the common underlying hazard given in (1) among those patients who are under observation at time t.

*λ_k_*(*t*; *Z_ik_*) = *λ*_0_(*t*)*e^β^ ′Zik* (*t*) (1)

PWP propose a stratified model for each event and consequently, each event has a different risk. In this case, it also considers that the process at risk for an event only happens if the previous one has happened (it is a conditional model). Specifically, they

*λ_k_*(*t*; *Z_ik_*) = *λ*_0_*_k_* (*t*)*e^β^ ′Zik* (*t*) (2)

where λ0k(t) is the baseline hazard for each event and Zik is a covariate vector of p-dimension for the ith subject at the kth event. In both models, β represents the vector of regression coefficients. Specifically, the hazard ratio (HR) for a covariate is calculated as the eβ.

The choice of an appropriate model for the time to recurrent infections depends on the process of the disease. If the risk of subsequent infections changes after the first infection, a suitable model should incorporate a time-dependent covariate for the number of infections (AG model adjusted by this variable) or use a model with separate strata for each event (PWP model). On the other hand, if the risk of recurrent infections remains constant regardless of previous infections, an AG model without adjusting for the time-dependent variable would be appropriate. In this model, follow-up time starts from the date of inclusion of each participant in the study.

To address the issue of potential inflation of type I errors caused by multiple observations per individual, we applied the sandwich robust standard error method introduced by Lin and Wei [3, 4]. This variance-correction technique was used in conjunction with the Cox extended models and does not require the specification of the correlation matrix, ensuring reliable and accurate results in the presence of recurrent events.

To investigate the dependence among recurrent times, we created a time-dependent variable that encoded the number of infections into the AG model. The results showed that the risk of infection decreased as the number of prior infections increased. Since the AG model assumes a common baseline risk function for all events, we concluded that using a stratified model (PWP model) per number of infections or an AG model adjusted for the time-dependent variable is equally suitable.

We fitted an AG model adjusted by sociodemographic information (sex, age as a 10-year increase, and job title), number of chronic conditions, SARS-CoV-2 vaccines (number of doses), and number of infections. Vaccines and infections are time-dependent variables. Additionally, we adjusted two PWP models: one for subjects without or with one infection, and another model for subjects with two or three infections, both adjusting with the same covariates as the AG model, except for the number of infections. The models incorporated data spanning from the inclusion date, which ranged from March 3, 2020, to March 22, 2021, up to December 31, 2022.

Statistical tests were performed with bilateral contrasts and statistical significance of P < .05. The analyses were carried out with the R package version 4.1.2 (R development Core Team, GNU, GPL) and RStudio version 1.4.1106 (R Foundation for Statistical Computing, Vienna, Austria).

**References**

1. Andersen, P.K., Gill, R.D.: Cox’s regression model for counting processes: A large

sample study. The Annals of Statistics 10 (2007) https://doi.org/10.1214/aos/

1176345976

2.Prentice, R.L., Williams, B.J., Peterson, A.V.: On the regression analysis of multivariate

failure time data. Biometrika 68 (1981) https://doi.org/10.1093/biomet/

68.2.373

3 Therneau, T.M., Grambsch, P.M. Modeling Survival Data: Extending the Cox Model. Springer, New York, 2000.

4 Lin, D.Y.,Wei, L.J.: The robust inference for the cox proportional hazards model.

Journal of the American Statistical Association 84, 1074–1078 (1989) https://

doi.org/10.1080/01621459.1989.10478874

**Additional results**

**Table S1** Analysis of SARS-CoV-2 infection risk: Andersen and Gill (AG) model adjusted by sex, age, job title, number of chronic conditions, vaccine doses, and infections.

*Note:* The model provides a comprehensive estimation of the factors of interest. Given the model’s use of a common baseline risk function, accounting for a time-dependent variable (number of infections) is imperative for studying the dependence among recurrent events. Significance levels were reported as: * for P ≤.05; ** for P ≤.01; and *** for P ≤.001. SE stands for standard error. The number of chronic diseases were calculated using the operational definition of the SNAC-K study.

| Predictor | HR | Robust SE | z-score | p-value |
| --- | --- | --- | --- | --- |
| Sex (ref = Female) |  |  |  |  |
| Male | 1.12 | 0.08 | 1.31 | .19 |
| Age-10 years increase | 0.94 | 0.04 | -1.50 | .13 |
| Job title (ref = Physician) |  |  |  |  |
| Nurse | 1.12 | 0.10 | 1.11 | .27 |
| Nurse assistant | 1.17 | 0.14 | 1.09 | .27 |
| Physiotherapist | 0.61 | 0.32 | -1.53 | .13 |
| Administrative staff | 0.88 | 0.16 | -0.82 | .41 |
| Social worker | 0.76 | 0.31 | -0.90 | .37 |
| Others | 0.79 | 0.13 | -1.89 | .06 |
| Number of chronic conditions (ref = 0-1) |  |  |  |  |
| 2-3 | 1.18 | 0.09 | 1.85 | .06 |
| >3 | 1.19 | 0.09 | 1.96 | .050 |
| Number of doses (ref = 0) |  |  |  |  |
| 1 | 0.32 | 0.24 | -4.82 | <.001 |
| 2 | 0.19 | 0.18 | -9.23 | <.001 |
| 3 | 0.10 | 0.17 | -13.62 | <.001 |
| 4 | 0.01 | 0.42 | -11.11 | <.001 |
| Number of infections | 0.26 | 0.13 | -10.60 | <.001 |
|  |  |  |  |  |

**Table S2** Analysis of SARS-CoV-2 infection risk using two Prentice, Williams, and Peterson models: the first one includes subjects without or with only one infection, and the other the subjects with two or three infections.

*Note:* Significance levels were reported as: * for P ≤.05; ** for P ≤.01; and *** for P ≤.001. SE stands for standard error. The number of chronic diseases were calculated using the operational definition of the SNAC-K study.

|  |  |  |  |  |  |  |  |  |  |
| --- | --- | --- | --- | --- | --- | --- | --- | --- | --- |
| PWP model | With 0 or 1 infection | | | | | With 2 or 3 infections | | | |
| Predictor | HR | Robust SE | z-score | p-value |  | HR | Robust SE | z-score | p-value |
| Sex (ref = Female) |  |  |  |  |  |  |  |  |  |
| Male | 1.22 | 0.08 | 2.43 | .02 | *. | 0.86 | 0.18 | -0.85 | .39 |
| Age-10 years increase | 0.96 | 0.05 | -0.74 | .46 |  | 0.91 | 0.08 | -1.26 | .21 |
| Job title (ref = Physician) |  |  |  |  |  |  |  |  |  |
| Nurse | 1.09 | 0.12 | 0.77 | .44 |  | 1.19 | 0.18 | 0.97 | .33 |
| Nurse assistant | 1.24 | 0.15 | 1.45 | .15 |  | 0.91 | 0.27 | -0.35 | .72 |
| Physiotherapist | 0.96 | 0.47 | -0.09 | .93 |  | 0.00 | 0.68 | -22.96 | <.001 |
| Administrative staff | 0.89 | 0.21 | -0.55 | .58 |  | 0.77 | 0.25 | -1.04 | .30 |
| Social worker | 0.91 | 0.37 | -0.26 | .80 |  | 0.32 | 0.90 | -1.28 | .20 |
| Others | 1.15 | 0.13 | 1.08 | .28 |  | 0.34 | 0.35 | -3.09 | .002 |
| Number of chronic conditions (ref = 0-1) |  |  |  |  |  |  |  |  |  |
| 2-3 | 1.41 | 0.09 | 3.86 | <.001 | *** | 0.87 | 0.18 | -0.79 | .43 |
| >3 | 1.39 | 0.10 | 3.19 | .001 | ** | 0.73 | 0.23 | -1.40 | .16 |
| Number of doses (ref = 0) |  |  |  |  |  |  |  |  |  |
| 1 | 0.32 | 0.45 | -2.54 | .01 | *. | 0.97 | 0.46 | -0.06 | .96 |
| 2 | 0.13 | 0.26 | -8.05 | <.001 | *** | 0.86 | 0.42 | -0.37 | .71 |
| 3 | 0.07 | 0.23 | -11.65 | <.001 | *** | 0.56 | 0.42 | -1.37 | .17 |
| 4 | 0.01 | 0.51 | -9.40 | <.001 | *** | 0.03 | 0.82 | -4.42 | <.001 |
|  |  |  |  |  |  |  |  |  |  |
