# Supplementary material for: SARS-CoV-2 Infection Risk by Vaccine Doses and Prior Infections Over 24 Months: ProHEpiC-19 Longitudinal Study
Source: JMIR Public Health Surveill. 2024 Nov 22;10:e56926. doi: 10.2196/56926 (PMC11606241; doi:10.2196/56926)
Supplement: Multimedia Appendix 2 [file publichealth-v10-e56926-s002.docx]

**List of ProHEpiC-19 Investigators:**

Magda Alemany Costa (Unitat de Suport a la Recerca Metropolitana Nord, Institut Universitari d’Investigació en Atenció Primària Jordi Gol [IDIAP Jordi Gol], Mataró, Spain); Lucía A Carrasco-Ribelles (Unitat de Suport a la Recerca Metropolitana Nord, Institut Universitari d’Investigació en Atenció Primària Jordi Gol [IDIAP Jordi Gol], Mataró, Spain); Carla Chacón (Unitat de Suport a la Recerca Metropolitana Nord, Institut Universitari d’Investigació en Atenció Primària Jordi Gol [IDIAP Jordi Gol], Mataró, Spain); Anna Costa-Garrido (Unitat de Suport a la Recerca Metropolitana Nord, Institut Universitari d’Investigació en Atenció Primària Jordi Gol [IDIAP Jordi Gol], Mataró, Spain); Gala Diez Fadrique (Unitat de Suport a la Recerca Metropolitana Nord, Institut Universitari d’Investigació en Atenció Primària Jordi Gol [IDIAP Jordi Gol], Mataró, Spain); Rosalia Dacosta-Aguayo (Unitat de Suport a la Recerca Metropolitana Nord, Institut Universitari d’Investigació en Atenció Primària Jordi Gol [IDIAP Jordi Gol], Mataró, Spain); Rosa Garcia-Sierra (Unitat de Suport a la Recerca Metropolitana Nord, Institut Universitari d’Investigació en Atenció Primària Jordi Gol [IDIAP Jordi Gol], Mataró, Spain); Noemi Lamonja-Vicente (Unitat de Suport a la Recerca Metropolitana Nord, Institut Universitari d’Investigació en Atenció Primària Jordi Gol [IDIAP Jordi Gol], Mataró, Spain); Josep M Manresa-Dominguez (Unitat de Suport a la Recerca Metropolitana Nord, Institut Universitari d’Investigació en Atenció Primària Jordi Gol [IDIAP Jordi Gol], Mataró, Spain); Xaviera Molina (Unitat de Suport a la Recerca Metropolitana Nord, Institut Universitari d’Investigació en Atenció Primària Jordi Gol [IDIAP Jordi Gol], Mataró, Spain); Pilar Montero-Alia (Unitat de Suport a la Recerca Metropolitana Nord, Institut Universitari d’Investigació en Atenció Primària Jordi Gol [IDIAP Jordi Gol], Mataró, Spain); Ester Moral-Roldan (Unitat de Suport a la Recerca Metropolitana Nord, Institut Universitari d’Investigació en Atenció Primària Jordi Gol [IDIAP Jordi Gol], Mataró, Spain); Alba Pachón Camacho (Unitat de Suport a la Recerca Metropolitana Nord, Institut Universitari d’Investigació en Atenció Primària Jordi Gol [IDIAP Jordi Gol], Mataró, Spain); Marta Soldevilla Garcia (Unitat de Suport a la Recerca Metropolitana Nord, Institut Universitari d’Investigació en Atenció Primària Jordi Gol [IDIAP Jordi Gol], Mataró, Spain); Pere Torán-Monserrat (Unitat de Suport a la Recerca Metropolitana Nord, Institut Universitari d’Investigació en Atenció Primària Jordi Gol [IDIAP Jordi Gol], Mataró, Spain); Concepción Violán (Unitat de Suport a la Recerca Metropolitana Nord, Institut Universitari d’Investigació en Atenció Primària Jordi Gol [IDIAP Jordi Gol], Mataró, Spain); Marc Boigues (Immunology Division, Laboratori Clinic Metropolitana Nord [LCMN], Hospital Universitari Germans Trias i Pujol [HUGTiP], Badalona, Spain); Bibiana Quirant (Immunology Division, Laboratori Clinic Metropolitana Nord [LCMN], Hospital Universitari Germans Trias i Pujol [HUGTiP], Badalona, Spain); Eva Martínez-Cáceres (Immunology Division, Laboratori Clinic Metropolitana Nord [LCMN], Hospital Universitari Germans Trias i Pujol [HUGTiP], Badalona, Spain); Inés Vanhille (Immunology Division, Laboratori Clinic Metropolitana Nord [LCMN], Hospital Universitari Germans Trias i Pujol [HUGTiP], Badalona, Spain). Laia Bernard (IrsiCaixa-AIDS Research Institute, Badalona, Spain); Bonaventura Clotet (IrsiCaixa-AIDS Research Institute, Badalona, Spain); Julia G Prado (IrsiCaixa-AIDS Research Institute, Badalona, Spain); Eulalia Grau Segura (IrsiCaixa-AIDS Research Institute, Badalona, Spain); Ruth Peña Poderós (IrsiCaixa-AIDS Research Institute, Badalona, Spain); Raul Pérez-Caballero (IrsiCaixa-AIDS Research Institute, Badalona, Spain); Gabriel Felipe Rodriguez-Lozano (IrsiCaixa-AIDS Research Institute, Badalona, Spain); M José Argerich González (Unitat de Suport a la Recerca Metropolitana Nord, Institut Universitari d’Investigació en Atenció Primària Jordi Gol [IDIAP Jordi Gol], Mataró, Spain; Ester Badia (Unitat de Suport a la Recerca Metropolitana Nord, Institut Universitari d’Investigació en Atenció Primària Jordi Gol [IDIAP Jordi Gol], Mataró, Spain; Josep Ma Bonet Simó (Unitat de Suport a la Recerca Metropolitana Nord, Institut Universitari d’Investigació en Atenció Primària Jordi Gol [IDIAP Jordi Gol], Mataró, Spain; Anna Devesa Pradells (Unitat de Suport a la Recerca Metropolitana Nord, Institut Universitari d’Investigació en Atenció Primària Jordi Gol [IDIAP Jordi Gol], Mataró, Spain); Elena Domenech Graells(Unitat de Suport a la Recerca Metropolitana Nord, Institut Universitari d’Investigació en Atenció Primària Jordi Gol [IDIAP Jordi Gol], Mataró, Spain); Nemesio Moreno Millán (Unitat de Suport a la Recerca Metropolitana Nord, Institut Universitari d’Investigació en Atenció Primària Jordi Gol [IDIAP Jordi Gol], Mataró, Spain); Eduard Moreno Gabriel (Unitat de Suport a la Recerca Metropolitana Nord, Institut Universitari d’Investigació en Atenció Primària Jordi Gol [IDIAP Jordi Gol], Mataró, Spain); Alex Ortega Roca (Unitat de Suport a la Recerca Metropolitana Nord, Institut Universitari d’Investigació en Atenció Primària Jordi Gol [IDIAP Jordi Gol], Mataró, Spain); Mónica Piña Rodriguez (Unitat de Suport a la Recerca Metropolitana Nord, Institut Universitari d’Investigació en Atenció Primària Jordi Gol [IDIAP Jordi Gol], Mataró, Spain); Nuria Prat Gil (Unitat de Suport a la Recerca Metropolitana Nord, Institut Universitari d’Investigació en Atenció Primària Jordi Gol [IDIAP Jordi Gol], Mataró, Spain); Victòria Sabaté Cintas (Unitat de Suport a la Recerca Metropolitana Nord, Institut Universitari d’Investigació en Atenció Primària Jordi Gol [IDIAP Jordi Gol], Mataró, Spain); Asumció Vázquez Durán (Unitat de Suport a la Recerca Metropolitana Nord, Institut Universitari d’Investigació en Atenció Primària Jordi Gol [IDIAP Jordi Gol], Mataró, Spain).
